# Integrative osmotic–antioxidant mechanisms in salinity-stressed *Gerbera jamesonii* treated with proline–IAA and PGPR

**DOI:** 10.3389/fpls.2025.1733697

**Published:** 2026-01-12

**Authors:** Ümmü Özgül Karagüzel

**Affiliations:** Department of Horticulture, Faculty of Agriculture, Recep Tayyip Erdoğan University, Rize, Türkiye

**Keywords:** bacterial inoculation, chlorophyll stability, *Gerbera jamesonii* L., oxidative stress response, physiological tolerance, proline–indole-3-acetic acid (IAA), salinity stress

## Abstract

Salinity stress is a major abiotic constraint that adversely affects the productivity and ornamental value of *Gerbera jamesonii* Bolus ex Hook. f. This study evaluated the physiological and biochemical responses of two cultivars (‘Zingana’ and ‘Sene Vidi’) to NaCl-induced salinity and examined the mitigation potential of microbial and biostimulant applications. Plants were subjected to six treatments under controlled greenhouse conditions: Control, NaCl, NaCl + Bacteria, NaCl + Proline–IAA, Bacteria, and Proline–IAA. Salinity markedly reduced relative water content (RWC), chlorophyll pigments, and leaf area, while increasing lipid peroxidation (MDA), indicating oxidative and osmotic stress. The bacterial and Proline–IAA treatments, applied either alone or under salinity, alleviated these negative effects by maintaining water status, stabilizing pigments, and lowering MDA levels. The Proline–IAA biostimulant, in particular, enhanced osmotic regulation and chlorophyll preservation, while bacterial inoculation improved overall physiological resilience. Principal component and correlation analyses revealed strong positive associations among RWC, chlorophyll content, and leaf area, whereas MDA was negatively correlated with growth parameters. Overall, both microbial and Proline–IAA applications improved the salinity tolerance of *G. jamesonii*, supporting their use as sustainable tools for maintaining growth performance and ornamental quality under saline conditions.

## 1 Introduction

*Gerbera jamesonii* L. Bolus is among the five most commercially valuable cut flowers worldwide, appreciated for its striking color diversity, long vase life, and year-round demand. According to Royal FloraHolland (2020), *Gerbera* consistently ranks within the top five traded cut flowers, with annual sales exceeding one billion stems and generating over €130 million in revenue. The global cut-flower industry continues to expand and is expected to grow from USD 39.08 billion in 2024 to USD 51.83 billion by 2030 (Grand View Research, 2025). Despite this strong market performance, *G. jamesonii* remains highly sensitive to salinity, which compromises growth and diminishes aesthetic quality under greenhouse conditions. Elevated electrical conductivity (EC) in the root zone reduces flower yield, chlorophyll content, and relative water content (RWC) (Saraçoğlu et al., 2019; Uzma et al., 2022a).

Salinity is widely recognized as one of the most damaging abiotic stresses, leading to osmotic imbalance, ion toxicity, and oxidative injury that collectively suppress photosynthesis and water uptake (Munns and Tester, 2008; Gill and Tuteja, 2010). In ornamental species, the degree of salt sensitivity varies greatly, often reflected by changes in pigment composition, ion accumulation, and visible quality traits (Wang et al., 2025). In *Gerbera* and other floricultural crops, NaCl exposure accelerates pigment degradation and membrane lipid peroxidation, commonly indicated by increased malondialdehyde (MDA) levels (Hnilickova et al., 2021; Xu et al., 2021). Comparable oxidative trends have been observed in *Tagetes* and *Amaranthus*, where salinity elevated MDA but decreased chlorophyll pigments (Hossain et al., 2022; Chrysargyris et al., 2018).

Among the physiological adjustments, proline accumulation plays a pivotal role in osmotic regulation and the stabilization of cellular structures (Szabados and Savouré, 2010; Ashraf and Foolad, 2007; Hayat et al., 2012). Exogenous application of proline has repeatedly been reported to alleviate salt-induced damage by sustaining pigment integrity and RWC in both ornamentals and vegetable crops, including *Gerbera* and *Solanum lycopersicon* (El-Banna and Mosa, 2024; Nakhaie et al., 2022). Likewise, Farooq et al. (2025) observed that supplemental proline improved salt acclimation in soybean by modulating osmolyte metabolism through the ornithine–glutamate dual pathway, suggesting its involvement in ion balance and stress signaling. Similar osmolyte-related regulation has been reported in barley under saline conditions (Boussora et al., 2024).

Salinity also alters photosynthetic pigment profiles. Typically, chlorophyll a, chlorophyll b, and total chlorophyll decline, while carotenoids tend to accumulate owing to their photoprotective and antioxidant functions (García-Caparrós and Lao, 2018; Kumara et al., 2021). Light intensity can further influence these responses; Fitzner et al. (2023) demonstrated that pigment content in halophytes shifts depending on the interaction between salinity and irradiance. Interestingly, some ornamentals such as *Tagetes* show increased antioxidant activity despite reduced floral size under saline conditions (Guzman and Marques, 2023).

Beyond pigments and osmolytes, secondary metabolites particularly phenolic compounds play an essential role in non-enzymatic antioxidant defense. Their accumulation contributes to the detoxification of reactive oxygen species (ROS) and limits lipid peroxidation, thereby protecting cellular membranes (Bayat et al., 2022; Toscano et al., 2023). Comparable antioxidant dynamics have been reported in *Pistacia vera* rootstocks (Rahneshan et al., 2018). Relative water content (RWC) remains a sensitive indicator of plant hydration and an integrative measure of turgor maintenance under stress (Uzma et al., 2023). Although many studies have examined individual parameters under saline conditions, comprehensive evaluations that integrate oxidative markers (MDA), osmolyte accumulation (proline), pigment stability (chlorophylls and carotenoids), phenolic metabolism, and water relations (RWC) are still scarce for *Gerbera*.Recent bibliometric evidence further confirms that abiotic stress research in ornamental plants—including Gerbera—remains considerably underrepresented compared to edible crops, despite increasing scientific interest in stress physiology (Karagüzel, 2025). This highlights the need for comprehensive, multi-parameter evaluations in floricultural species to better understand and manage stress responses.

In light of these gaps, the present study aimed to assess the effectiveness of exogenous proline supplemented with indole-3-acetic acid (IAA) and plant growth-promoting bacteria (*Bacillus megaterium*, *Paenibacillus polymyxa*, *Pantoea agglomerans*, and *Pseudomonas fluorescens*) in mitigating salinity stress in two *Gerbera* cultivars. We hypothesized that these treatments would alleviate salt-induced injury by enhancing osmotic adjustment, preserving pigment stability, and reinforcing non-enzymatic antioxidant capacity. Importantly, this work provides the first integrative evaluation of proline- and PGPR-mediated mitigation of salinity in *Gerbera jamesonii* at combined morphological, physiological, and biochemical levels, thereby addressing a critical knowledge gap in ornamental stress physiology.

## 2 Materials and methods

### 2.1 Plant material and growth conditions

The experiment was conducted in the greenhouse facilities of the Faculty of Agriculture, Recep Tayyip Erdoğan University (Rize, Türkiye) under controlled environmental conditions during July. The 60 m^2^ polycarbonate greenhouse maintained average daytime temperatures of 24–28°C and nighttime temperatures of 18–20°C, with 70–80% relative humidity and a natural photoperiod of approximately 14–15 h.Two commercial cultivars of *Gerbera jamesonii* L. ‘Zingana’ (red flowers) and ‘Sene Vidi’ (white flowers)—were grown in 0.5 L plastic pots filled with a sand:peat mixture (1:2, v/v) having an initial electrical conductivity (EC) of 1.2–1.5 dS m^−1^ and a pH between 5.5 and 6.2. Before treatment application, plants were irrigated with tap water and fertilized weekly with a balanced 20:20:20 NPK solution (150 mg L^−1^). Salinity stress was initiated when plants reached the early vegetative stage (4–5 fully expanded leaves), recognized as a sensitive phase for salinity evaluation (Acosta-Motos et al., 2017).

### 2.2 Experimental design and treatments

The study followed a completely randomized design (CRD) with six treatments and three replications. Each replicate included three plants, making a total of 54 plants (6 × 3 × 3). Treatments were as follows: (1) Control (C) without salinity stress, (ii) NaCl (S) applied at 150 mM, (iii) Proline–IAA (P) consisting of a commercial formulation containing L-proline and indole-3-acetic acid (IAA) (Supersol^®^, Türkiye), (iv) PGPR (B) inoculation using a microbial biostimulant containing *Bacillus megaterium*, *Paenibacillus polymyxa*, *Pantoea agglomerans*, and *Pseudomonas fluorescens* (Supersol^®^, Türkiye), (v) the combined NaCl + Proline–IAA (S + P) treatment, and (vi) the combined NaCl + PGPR (S + B) treatment. Salinity was induced with 150 mM NaCl, applied in two steps (75 mM per day) to avoid osmotic shock. The concentration of 150 mM NaCl was selected based on previous studies showing that this level induces a strong salinity stress in *Gerbera jamesonii* without causing immediate plant collapse. In particular, Zulfiqar et al. (2023) and Uzma et al. (2022a) demonstrated that gerbera cultivars exposed to 150 mM NaCl exhibit marked oxidative damage, pigment loss, and clear reductions in physiological performance. Moreover, a recent review by Toscano et al. (2023) highlights that NaCl concentrations within the 100–200 mM range are widely used to impose reproducible salinity stress in sensitive ornamental species. The Proline–IAA treatment was applied as a commercial Supersol^®^ biostimulant containing L-proline and indole-3-acetic acid (IAA) at a dosage of 50 mL pot^−1^ (equivalent to 20 mM proline). The exact concentration of IAA is proprietary and not disclosed by the manufacturer; however, the formulation was applied strictly according to the recommended usage instructions. The PGPR treatment consisted of a rhizobacterial consortium (SS-Super Root^®^, Turkey) containing *Bacillus megaterium*, *Paenibacillus polymyxa*, *Pantoea agglomerans*, and *Pseudomonas fluorescens* at a density of 10^8^ CFU mL^−1^. For application, 50 mL of the commercial inoculum was mixed with 1 L of water to prepare the working solution, and this mixture was applied as a 10-mL soil drench per pot at the start of the experiment. As this product is a standardized proprietary formulation, the manufacturer does not disclose the relative strain ratios. The inoculum was applied once at the beginning of the experiment as a soil drench at 10 mL per plant, delivered directly to the root zone. No repeated applications or additional nutrient supplementation were used during the stress period. In the combined treatments, NaCl stress and biostimulant applications were imposed simultaneously.

### 2.3 Measurement and evaluation schedule

Observations were made at three growth stages Initial (Day 0), Intermediate (Day 14), and Final (Day 30) defined according to visible leaf symptoms and substrate EC. Measurements were conducted on Day 14, selected as an intermediate time point within the 2–3 week in which salinity-induced pigment, osmolytes, and oxidative responses have been reported in Gerbera jamesonii (Uzma et al., 2022a; Farooq et al., 2023; Cioć et al., 2022). Plant height and leaf number were recorded at the final stage, while biochemical traits (malondialdehyde [MDA], proline, total phenolics, and carotenoids) were measured at the intermediate stage. Physiological parameters (RWC, chlorophyll a, chlorophyll b, total chlorophyll, chlorophyll a/b ratio and leaf area) were assessed across all three stages to capture early and cumulative responses.

### 2.4 Morphological measurements

Plant height and leaf number were measured manually with a ruler and direct counts. Leaf area was determined using an image analysis system (WinDIAS 3, Delta-T Devices Ltd., Cambridge, UK) as described by Smart and Bingham (1974).

### 2.5 Physiological analysis

Relative water content (RWC, %) was determined following Weatherley (1950):


$$RWC=\frac{\left(FW-DW\right)}{\left(TW-DW\right)}\times 100$$

where FW = fresh weight, TW = turgid weight after 4 h rehydration, and DW = dry weight after drying at 70°C for 48 h.

### 2.6 Biochemical analyses

Malondialdehyde (MDA): Lipid peroxidation was estimated using the method of Heath and Packer (1968), modified by Kıran et al. (2022). Leaf samples (0.2 g) were homogenized in 5 mL of 0.1% (w/v) TCA and centrifuged at 12, 000 × g for 10 min at 4°C. The supernatant (2 mL) was mixed with 2 mL of 20% TCA containing 0.5% (w/v) TBA, incubated at 90°C for 30 min, cooled on ice, and centrifuged again. Absorbance was read at 532 and 600 nm (Shimadzu UV-1800, Japan). MDA concentration was calculated using ϵ = 155 mM^−1^ cm^−1^ and expressed as µmol g^−1^ FW.

Proline Content: Determined according to Bates et al. (1973). Leaf tissue (0.1 g) was homogenized in 3% sulfosalicylic acid, centrifuged at 10, 000 × g for 10 min, and the supernatant was reacted with acid ninhydrin reagent (2.5 g ninhydrin in 60 mL glacial acetic acid + 40 mL orthophosphoric acid). After incubation at 100°C for 30 min, the mixture was extracted with 5 mL toluene, and absorbance was recorded at 528 nm. Proline concentration was calculated from a standard curve.

Total Phenolic Content (TPC): Measured using the Folin–Ciocalteu method (Singleton and Rossi, 1965). Leaf extracts (0.2 mL) were mixed with 1.0 mL of diluted Folin reagent (1:10) and 0.8 mL of 7.5% Na_2_CO_3_. After 30 min in darkness, absorbance was read at 765 nm and expressed as mg gallic acid equivalents (GAE) g^−1^ FW.

### 2.7 Photosynthetic pigments

Chlorophyll a, chlorophyll b, total chlorophyll, chlorophyll a/b ratio, and carotenoids were quantified following Arnon (1949). Fresh leaf tissue (0.1 g) was homogenized in 5 mL of 80% acetone, centrifuged at 5, 000 rpm for 15 min at 4°C, and absorbance was measured at 663, 645, and 470 nm. Calculations followed the standard equations:


Chl a=12.7(A663)–2.69(A645);

Chl b=22.9(A645)–4.68(A663);

Carotenoids=[1000(A470)–1.82(Chl a)–85.02(Chl b)]/198.

### 2.8 Instrumentation

Spectrophotometric readings (MDA, proline, TPC, chlorophylls, and carotenoids) were obtained using a Shimadzu UV-1800 spectrophotometer (Kyoto, Japan) with 1-cm quartz cuvettes. Calibration was performed with appropriate blanks before each measurement.

### 2.9 Statistical analyses

Data were analyzed using a two-way ANOVA (treatment × time) in JMP Pro 17.0 (SAS Institute Inc., Cary, NC, USA). Assumptions of normality and homogeneity of variance were verified prior to analysis. For variables measured at multiple time points (leaf area, RWC, pigment traits, and biochemical parameters), the treatment × time ANOVA was performed separately for each cultivar because preliminary tests indicated significant cultivar effects. For single-time-point variables (plant height and leaf number), both cultivars were analyzed jointly with treatment as the main factor. When ANOVA indicated significant effects, mean separations were conducted using Tukey’s HSD test (p< 0.05).Pearson’s correlation coefficients were used to explore associations among traits. Multivariate relationships were further examined using principal component analysis (PCA) and hierarchical clustering (heatmap).

## 3 Results

This section describes the effects of salinity stress and mitigation treatments (proline and bacterial inoculation) on the morphological, physiological, and biochemical responses of *Gerbera jamesonii* cultivars. Statistical significance was determined using Tukey’s HSD test at p ≤ 0.05.

### 3.1 Effects of salinity and mitigation treatments on plant growth

**Figure 1:**
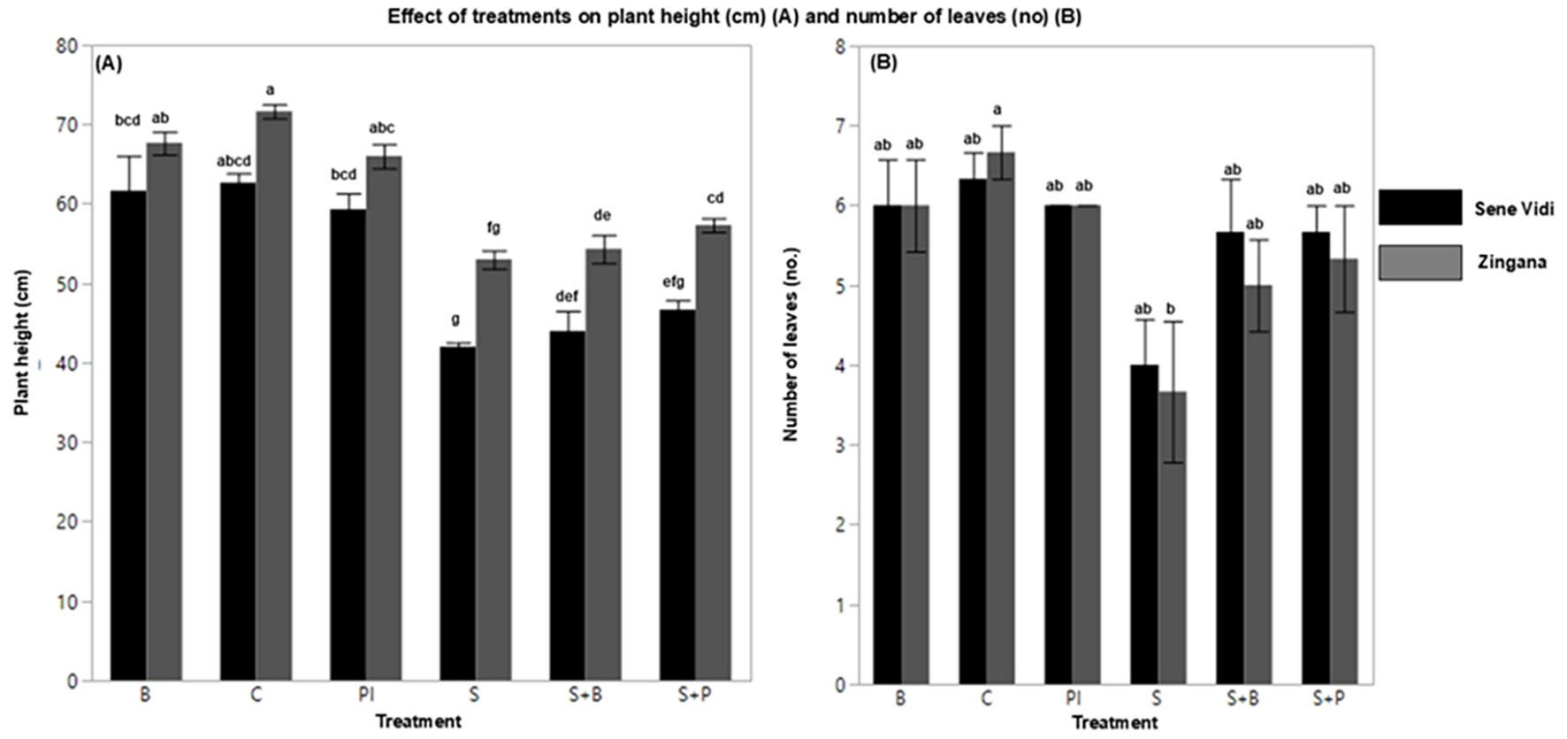
Mean (± SE, n = 9) values of morphological traits of *Gerbera jamesonii* under salinity and stress-relief treatments: **(A)** plant height (PH) and **(B)** number of leaves (NL). Control (C), Bacteria (B), Proline (P), NaCl (S), NaCl + Bacteria (S + B), NaCl + Proline (S + P), and NaCl + Proline-IAA (S + P). Black bars represent ‘Sene Vidi’ and gray bars represent ‘Zingana’.Different lowercase letters indicate significant differences among treatments (Tukey’s HSD, p< 0.05).

**Figure 2:**
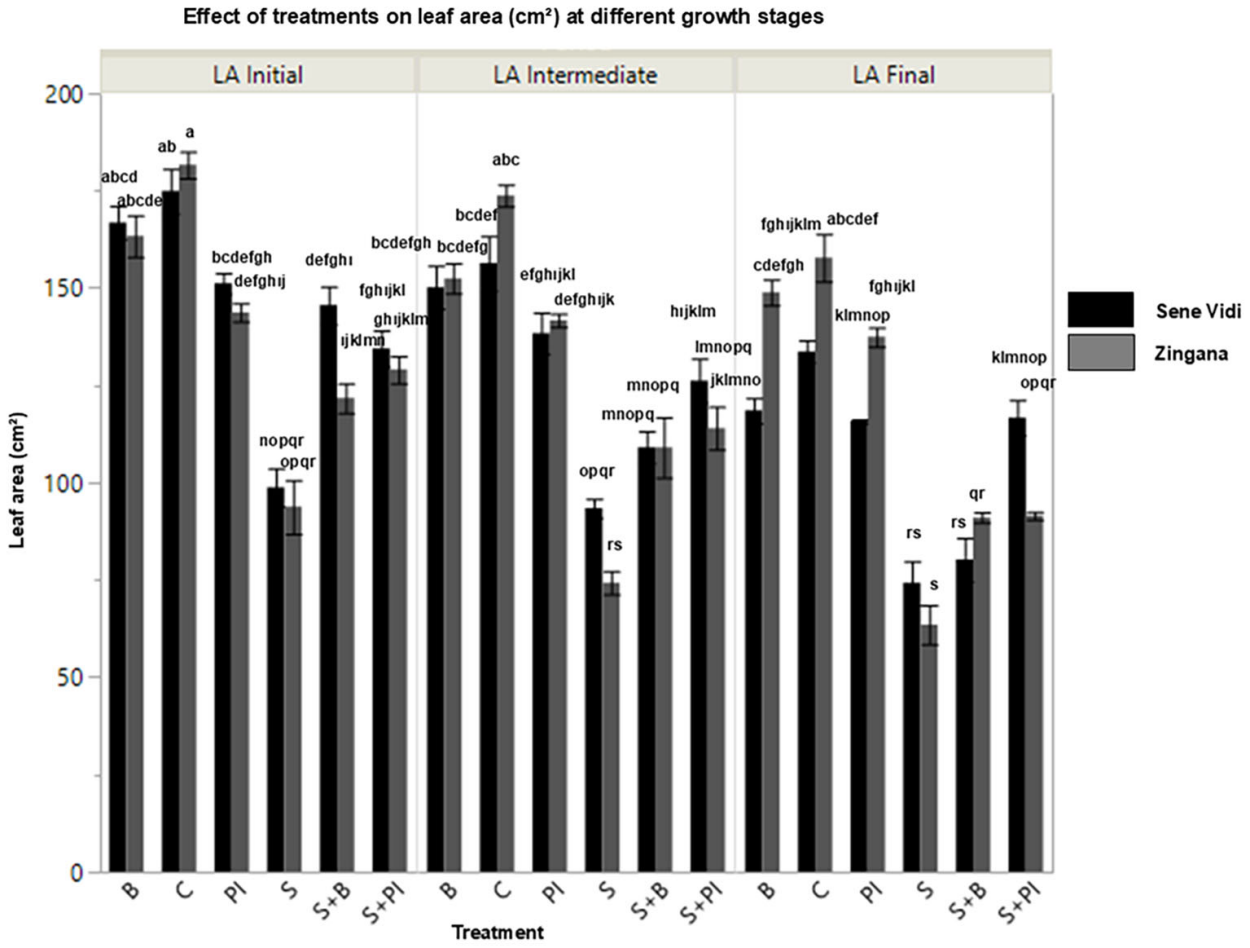
Leaf area (LA, cm^2^; mean ± SE; n = 9) of *Gerbera jamesonii* cultivars ‘Sene Vidi’ and ‘Zingana’ measured at three developmental stages (Initial, Intermediate, Final) under different treatments: Control (C), Bacteria (B), Proline (P), NaCl (S), NaCl + Bacteria (S + B), NaCl + Proline (S + P), and NaCl + Proline–IAA (S + PI). Black bars represent ‘Sene Vidi’ and gray bars represent ‘Zingana’. Each developmental stage is shown as a separate bar cluster from left to right (Initial, Intermediate, and Final). Different lowercase letters above bars indicate significant differences among treatments based on Tukey’s HSD test (p< 0.05).

Salinity stress significantly reduced plant height, leaf number, and leaf area in both cultivars and altered additional physiological and biochemical parameters, as shown in **Table 1** (**Figures 1**, **2**). **Table 1** also provides the mean ± SE values for each parameter, allowing clearer interpretation of the magnitude and practical relevance of treatment effects beyond the ANOVA statistics. Plants exposed to NaCl exhibited restricted leaf expansion and shortened shoots, indicating growth inhibition. In ‘Sene Vidi’, NaCl reduced plant height to ~40 cm, while control plants reached ~62–63 cm. In ‘Zingana’, NaCl reduced plant height to ~52–53 cm compared with ~70 cm in the control. Leaf number also decreased markedly in both cultivars, falling to ~4 leaves under NaCl compared with ~6–7 leaves in the controls.Exogenous proline and bacterial inoculation effectively alleviated these adverse effects and improved vegetative growth. In ‘Sene Vidi’, proline-treated plants reached ~59 cm, nearly matching control levels and exceeding the NaCl treatment by ~17–18 cm. Leaf number also increased to ~6 leaves compared with ~4 leaves under NaCl. Final-stage leaf area increased from ~70 cm^2^ (NaCl) to ~120–130 cm^2^ with proline.

**Table 1:** Analyses of variance for morphological, physiological, and biochemical parameters of *Gerbera jamesonii* under salinity and stress-relief treatments.

Parameter	PH	NL	LA	RWC	Chl *a*	Chl *b*	Chl *a+b*	Chl *a/b*	Carot	MDA	Pro	TPhenol
DF	11	11	11	11	11	11	11	11	11	11	11	11
Mean+SE	57.19 ± 1.60	5.52 ± 1.15	110.81 ± 5.03	77.66 ± 0.62	0.47 ± 0.009	0.21 ± 0.006	0.68 ± 0.01	2.35 ± 0.09	2.49 ± 0.07	1.90 ± 0.24	0.60 ± 0.03	3.85 ± 0.05
Mean square	270.755	2.452	2925.090	207.250	0.0412	0.0136	0.131	0.314	0.595	68.785	0.127	4.773
F-value	24.865	2.942	60.646	7.852	49.653	67.874	92.760	8.331	23.826	491.681	153.780	2.515
p-value	<0.0001	<0.0001	<0.0001	<0.0001	<0.0001	<0.0001	<0.0001	<0.0001	<0.0001	<0.0001	<0.001	<0.0001
Significance	*****	*	****	***	***	***	***	***	***	***	***	***

*, ***, **** and ***** indicate significance at p< 0.05, p< 0.01, p< 0.001, and p< 0.0001, respectively. PH, plant height (cm); NL, number of leaves; LA, leaf area (cm^2^); RWC, relative water content (%); *Chl* a, chlorophyll a (mg g^−1^ FW); *Chl* b, chlorophyll b (mg g^−1^ FW); *Chl* a + b, total chlorophyll (mg g^−1^ FW); *Chl* a/b, chlorophyll a/b ratio; Carot, carotenoids (mg g^−1^ FW); MDA, malondialdehyde (nmol g^−1^ FW); Pro, proline (µmol g^−1^ FW); TPhenol, total phenolic content (mg g^−1^ FW).

In ‘Zingana’, proline likewise enhanced growth, increasing final leaf area from ~53–58 cm^2^ (NaCl) to ~130–140 cm^2^. Bacterial inoculation produced moderate improvements, increasing plant height from ~52–53 cm (NaCl) to ~66–68 cm and leaf area from ~66–68 cm^2^ to ~140–145 cm^2^. These findings demonstrate that exogenous proline enhances vegetative development and preserves photosynthetic surface area under salinity stress in *Gerbera jamesonii*.

### 3.2 Relative water content

**Figure 3:**
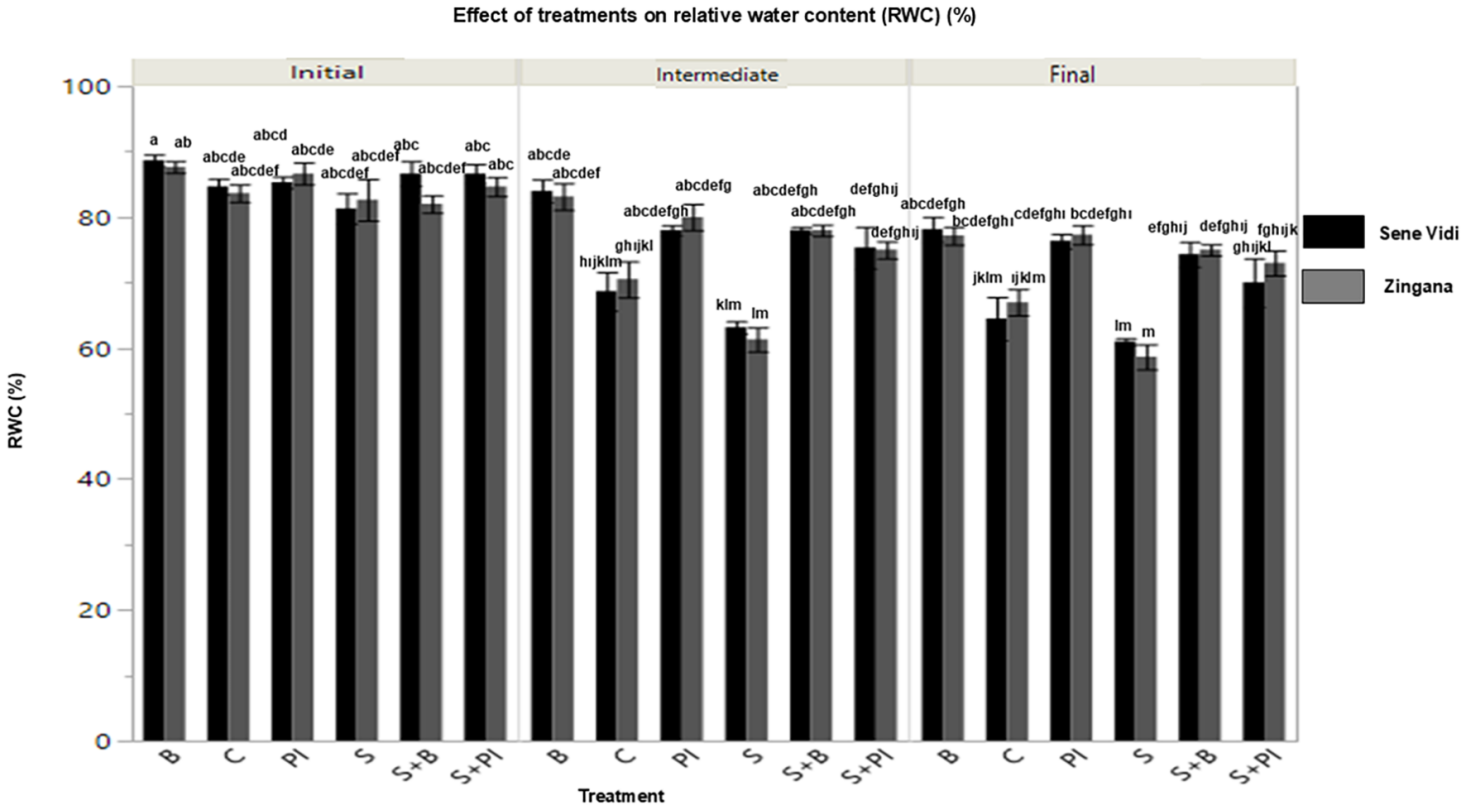
Relative water content (RWC, %) in *Gerbera jamesonii* cultivars ‘Sene Vidi’ and ‘Zingana’ at different time points (Initial, Intermediate, Final) under salinity and stress-relief treatments. Bars represent mean ± SE (n = 9). Control (C), Bacteria (B), Proline (P), NaCl (S), NaCl + Bacteria (S + B), NaCl + Proline (S + P), and NaCl + Proline -IAA (S + PI). Black bars represent ‘Sene Vidi’ and gray bars represent ‘Zingana’. Different lowercase letters above bars indicate significant differences among treatments based on Tukey’s HSD test (p< 0.05).

Salinity markedly decreased RWC compared with the control (**Figure 3**). The reduction was stronger in ‘Zingana’ than in ‘Sene Vidi’, indicating cultivar differences in water retention capacity.Both mitigation treatments increased RWC relative to the NaCl control. Proline consistently maintained the highest RWC across measurement periods, supporting its role in osmotic adjustment and membrane protection.Bacterial inoculation produced a delayed but positive effect, becoming significant in the final measurement. The NaCl + Proline IAA treatment sustained the greatest leaf hydration throughout the experiment, confirming its efficiency in maintaining water balance under salinity.

### 3.3 Photosynthetic pigment composition

**Figure 4:**
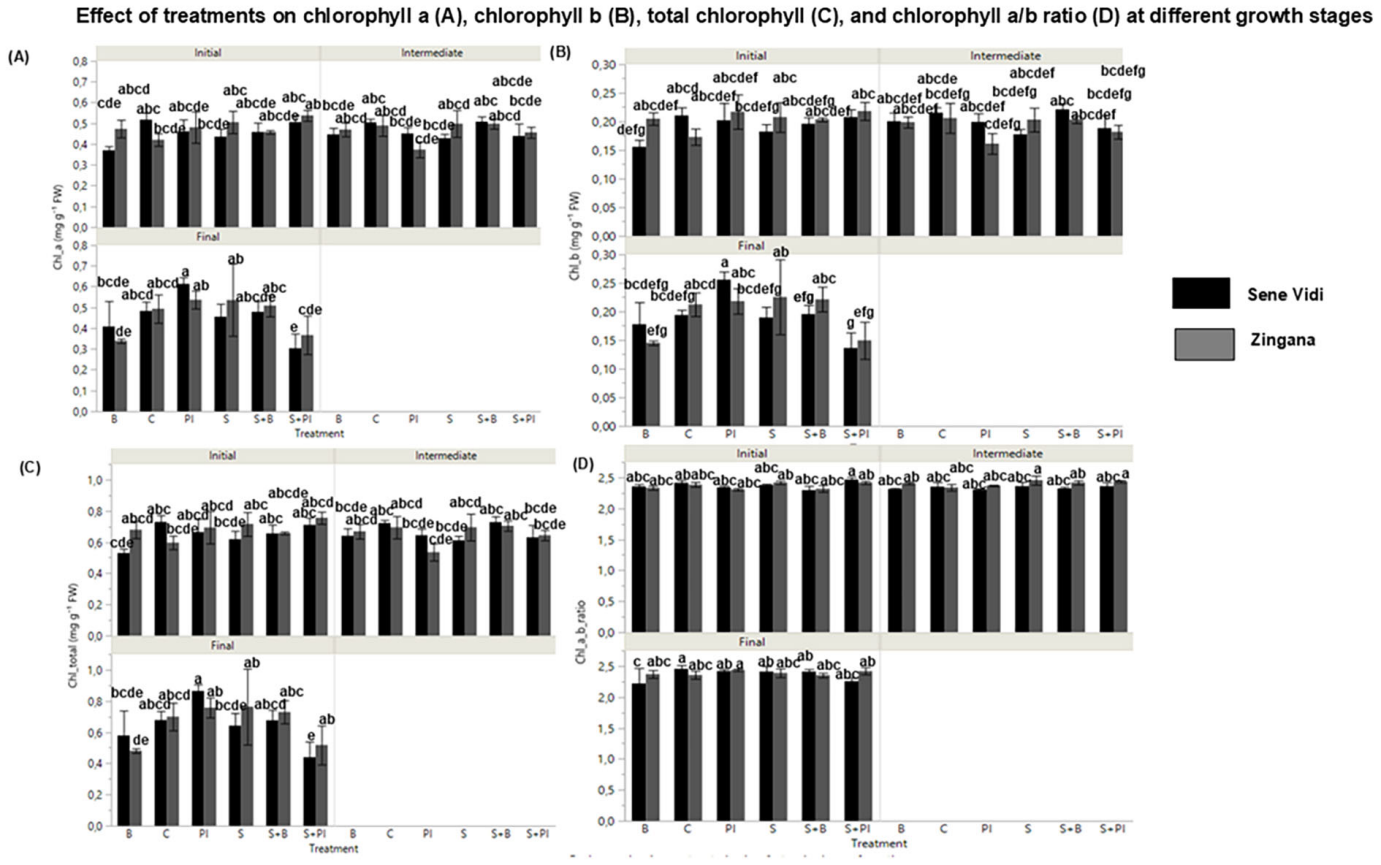
Mean (± SE, n = 9) values of **(A)** chlorophyll a, **(B)** chlorophyll b, **(C)** total chlorophyll, and **(D)** chlorophyll a/b ratio in *Gerbera jamesonii* cultivars under salinity and stress-relief treatments. Control (C), Bacteria (B), Proline (P), NaCl (S), NaCl + Bacteria (S + B), NaCl + Proline (S + P), and NaCl + Proline-IAA (S + PI). Black bars represent ‘Sene Vidi’ and gray bars represent ‘Zingana’. Each developmental stage is shown as a separate bar cluster from left to right (Initial, Intermediate, and Final).Black bars represent ‘Sene Vidi’ and gray bars represent ‘Zingana’. Different lowercase letters above bars indicate significant differences among treatments based on Tukey’s HSD test (p< 0.05).

Salinity significantly altered pigment composition (**Figure 4**). Both chlorophyll a and b decreased under NaCl stress, while the chlorophyll a/b ratio slightly increased, indicating preferential degradation of chlorophyll *b*. Proline-treated plants showed higher chlorophyll a, b, and total chlorophyll contents compared with salt-stressed plants. Bacterial inoculation produced moderate improvements, and the NaCl + Proline IAA treatment yielded the highest total chlorophyll concentrations. These results indicate that exogenous proline effectively preserves photosynthetic pigments and delays chlorophyll degradation under salinity stress in *Gerbera jamesonii*.

### 3.4 Oxidative stress indicators and osmoprotectant accumulation

**Figure 5:**
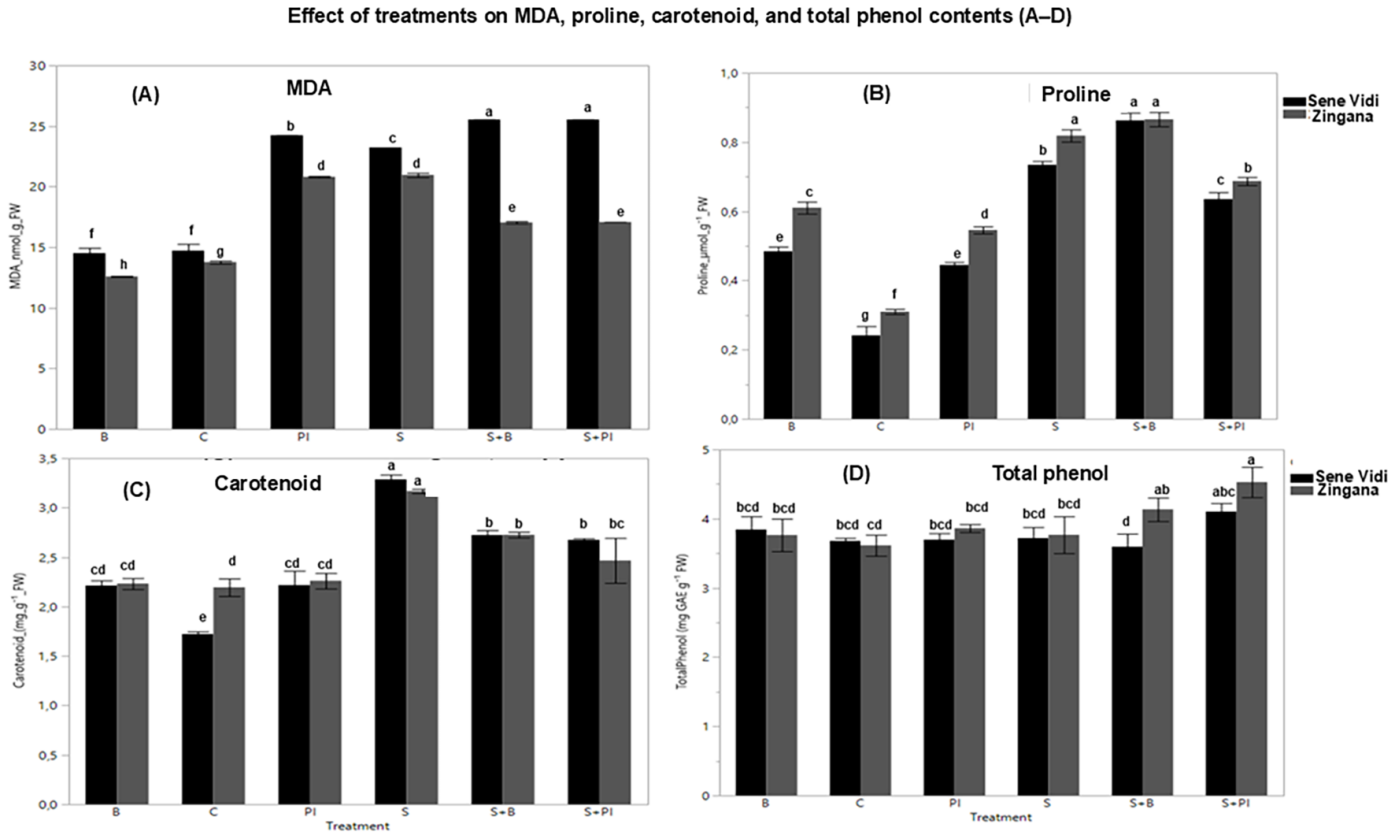
Mean (± SE, n = 9) values of **(A)** malondialdehyde (MDA), **(B)** proline, **(C)** carotenoids, and **(D)** total phenols in *Gerbera jamesonii* cultivars under salinity and stress-relief treatments. Control (C), Bacteria (B), Proline (P), NaCl (S), NaCl + Bacteria (S + B), NaCl + Proline (S + P), and NaCl + Proline-IAA (S + PI). Black bars represent ‘Sene Vidi’ and gray bars represent ‘Zingana’. Different lowercase letters above bars indicate significant differences among treatments based on Tukey’s HSD test (p< 0.05).

Salinity stress significantly increased malondialdehyde (MDA) content, confirming oxidative membrane injury (**Figure 5A**). Both proline and bacterial treatments reduced MDA levels compared with the salt-stressed control, with the NaCl + Proline IAA (S + PI) combination showing the strongest mitigation effect. Proline accumulation (**Figure 5B**) markedly increased under salinity and was further enhanced by exogenous application, indicating both uptake and induced synthesis. Bacterial inoculation also elevated proline, reflecting partial activation of osmoprotective metabolism. Carotenoids (**Figure 5C**) and total phenolics (**Figure 5D**) were significantly upregulated under mitigation treatments, highlighting their roles in non-enzymatic antioxidant defense. Overall, proline-IAA provided superior protection against oxidative stress, while bacterial inoculation contributed moderately by stimulating secondary antioxidant metabolism in *Gerbera jamesonii*.

### 3.6 Multivariate relationships among physiological and biochemical traits

To better understand the interrelationships among morphological, physiological, and biochemical parameters under salinity stress, Pearson correlation analysis and principal component analysis (PCA) were performed.

#### 3.6.1 Principal component analysis

**Figure 6:**
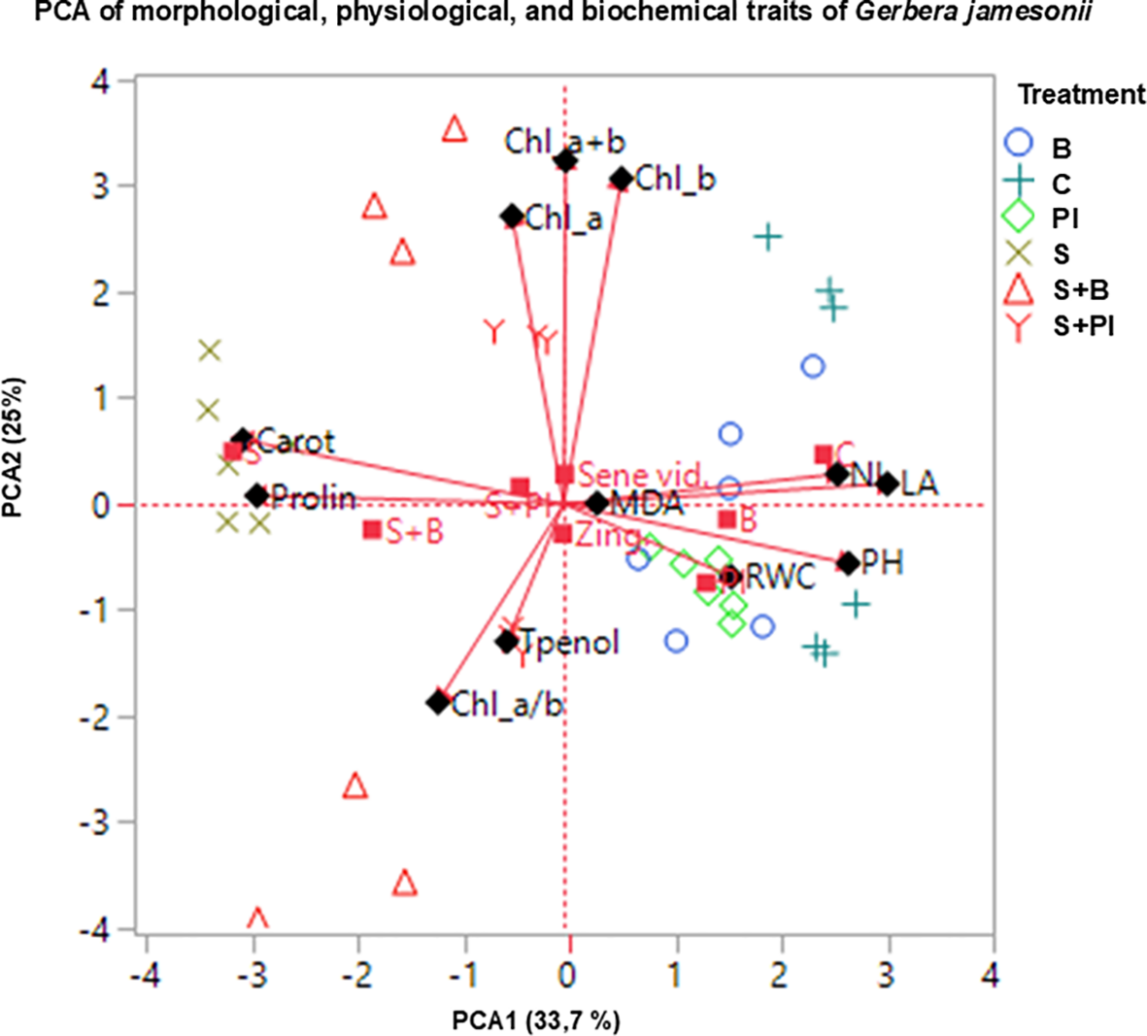
Principal component analysis (PCA) biplot showing the separation of treatments and their associations with physiological and biochemical traits of *Gerbera jamesonii* under salinity stress. PC1 and PC2 explain 33.7% and 25% of the total variance, respectively.

The PCA biplot (PC1 = 33.7% and PC2 = 25%, explaining a total of 58.7% of the variance) clearly separated treatments and visualized their associations with key physiological and biochemical traits (**Figure 6**). The first component (PC1) was mainly associated with morphological and pigment traits (PH, NL, LA, Chl a, Chl b, Chl a + b, RWC), while the second component (PC2) was dominated by biochemical parameters (MDA, proline, carotenoids, and total phenols). Treatments containing proline, particularly the NaCl + Proline IAA combination, clustered closely with chlorophyll fractions and RWC, indicating enhanced pigment stability and water balance under saline conditions. In contrast, NaCl-stressed plants were positioned near the MDA vector, reflecting increased oxidative injury and reduced physiological performance. The overall PCA pattern demonstrated that proline + IAA supplementation effectively mitigated salinity effects by coordinating osmotic adjustment and antioxidant protection mechanisms in *Gerbera jamesonii*.

#### 3.6.2 Correlation and heatmap analysis

**Figure 7:**
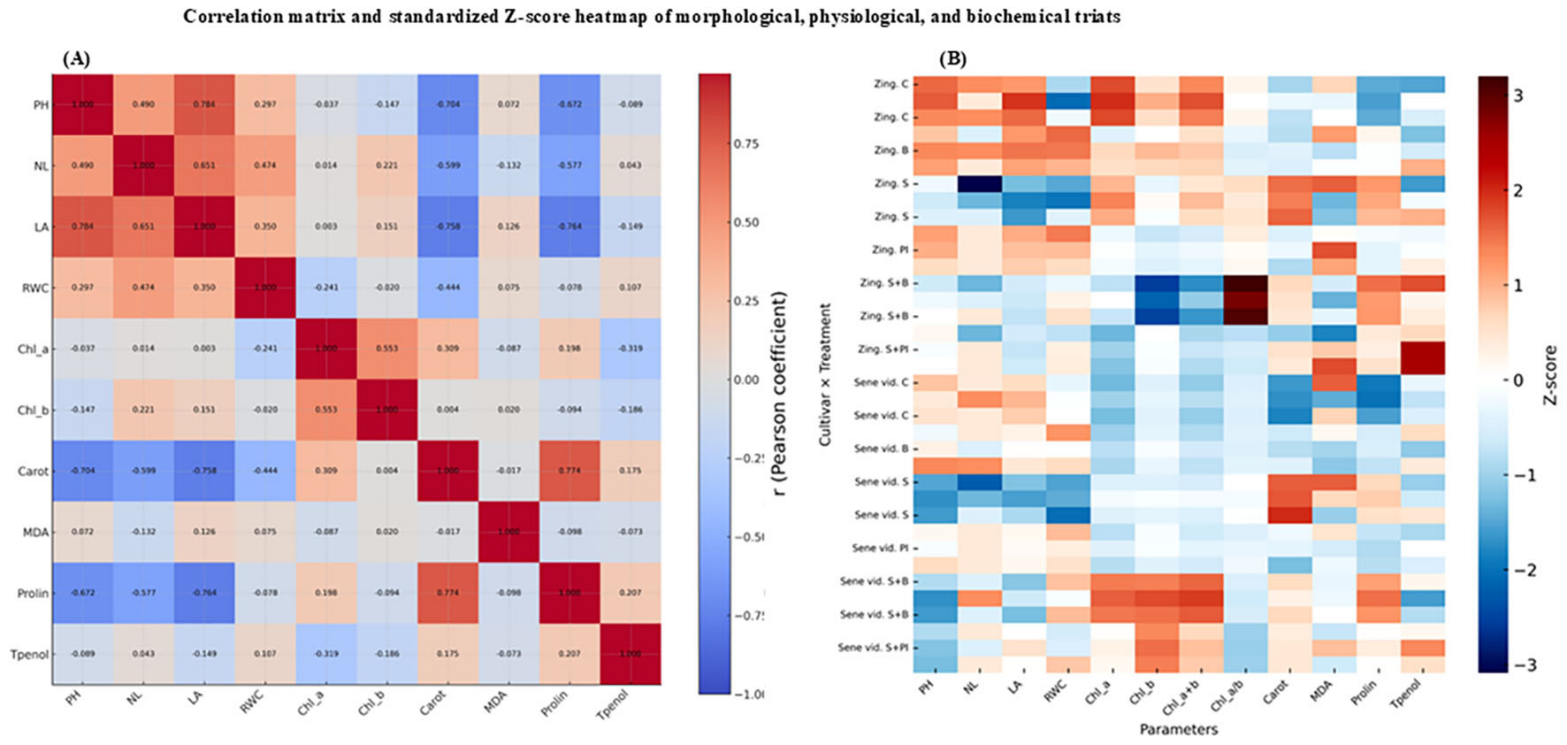
**(A)** Correlation matrix and **(B)** standardized Z-score heatmap of physiological and biochemical parameters of *Gerbera jamesonii* under salinity stress. Red and blue shades in panel **(A)** represent negative and positive correlations, respectively, whereas in panel **(B)** they indicate high and low standardized responses.

The correlation matrix (**Figure 7A**) revealed strong positive associations among growth (PH, NL, LA) and pigment parameters (Chl *a*, Chl *b*, total Chl). In contrast, MDA was negatively correlated with these traits, confirming its role as an indicator of oxidative stress. Carotenoids and phenols showed moderate positive correlations, suggesting coordinated activation of antioxidant metabolism. The standardized Z-score heatmap (**Figure 7B**) further supported these relationships, showing clear clustering of treatments based on stress intensity. The NaCl + Proline IAA treatment exhibited the most favorable physiological and biochemical profile, characterized by high chlorophyll, carotenoid, and phenolic contents alongside the lowest MDA levels.

## 4 Discussion

Salinity imposes complex physiological and biochemical constraints on plant systems by disturbing osmotic balance and enhancing oxidative stress. These disruptions adversely affect water relations, photosynthetic efficiency, and membrane stability, thereby compromising overall plant vigor. In the present study, *Gerbera jamesonii* exhibited typical salt-stress symptoms, including pigment loss and reduced leaf turgor, confirming its high sensitivity to NaCl. The application of stress-relief treatments, particularly Proline IAA and bacterial inoculation, improved tolerance by maintaining water status, protecting pigments, and limiting oxidative injury. The following sections discuss these coordinated responses through specific indicators such as MDA, RWC, pigments, and antioxidant metabolites.

### 4.1 Oxidative stress indicators (MDA)

Salinity stress induced a strong accumulation of malondialdehyde (MDA), indicating enhanced lipid peroxidation and oxidative injury. In this study, MDA levels increased by approximately 68% under NaCl treatment compared with the control, confirming oxidative membrane damage. Similar oxidative trends appeared in the PCA and heatmap analyses, where MDA vectors opposed RWC and chlorophyll fractions, highlighting negative correlations between oxidative stress and physiological performance. These results align with Don et al. (2010) and Hameed et al. (2021), who reported that prolonged salt exposure in *G. jamesonii* reduced chlorophyll and water balance via oxidative membrane injury. Recent reviews also emphasize that ROS overproduction under salinity triggers lipid peroxidation and photosynthetic inhibition (Liu et al., 2024; Zhao et al., 2021). Exogenous Proline IAA application, particularly in the S + PI treatment, reduced MDA by nearly 28% relative to the salt control, indicating osmolyte-assisted protection.

### 4.2 Proline accumulation and water relations (RWC)

Proline concentrations rose sharply under NaCl stress (approximately 2.3–2.6-fold compared with the control), confirming its central role in osmotic adjustment. The S + PI treatment maintained proline at approximately 2.0–2.3× control levels while supporting higher RWC, showing that exogenous supplementation enhanced endogenous accumulation and prevented excessive stress spikes. Bacterial inoculation and Proline IAA alone caused moderate increases, indicating a graded osmotic response. These findings correspond with Khedr et al. (2003) and Lacerda et al. (2020), who linked proline accumulation to upregulated stress proteins and improved leaf hydration. Mechanistically, proline contributes to ROS detoxification by modulating the glutathione–ascorbate cycle (Renzetti et al., 2024). PCA and correlation analyses further confirmed positive loadings of proline with RWC and negative associations with MDA, consistent with Zhao et al. (2021). Recent studies using different biostimulants further support this pattern. Silicon nanoparticles were reported to alleviate salinity-induced oxidative stress in Salvia officinalis by enhancing ROS scavenging and stabilizing membrane function (Shahpari et al., 2025). Likewise, melatonin improved drought tolerance in Taxus baccata by increasing osmolyte accumulation and antioxidant activity (Shahmohammadi et al., 2024), while methyl jasmonate reduced lipid peroxidation and promoted osmotic adjustment in Narcissus under salt stress (Dooz et al., 2024). These findings indicate that improved redox balance and osmotic regulation represent a shared mechanism across biostimulant strategies, aligning closely with the proline-mediated responses observed in Gerbera jamesonii.”.

### 4.3 Photosynthetic pigments (chlorophyll a, b, total chlorophyll, chlorophyll a/b ratio, and carotenoids)

NaCl stress caused marked reductions (approximately 30–35%) in chlorophyll a, chlorophyll b, and total chlorophyll, verifying the high pigment sensitivity of *G. jamesonii*. Partial pigment recovery under Proline IAA reflected osmolyte-mediated stabilization of chlorophyll–protein complexes and thylakoid membranes. Similar improvements were observed in *G. jamesonii* treated with salicylic acid (Farooq et al., 2025) and in *Chrysanthemum* (Vanlalruati et al., 2023). The concurrent rise in carotenoids (approximately 25–30%) under saline conditions and biostimulant treatments indicated enhanced photoprotection through singlet oxygen quenching, consistent with findings in *Tagetes*, *Pistacia vera*, and SiO_2_-NP-treated *Gerbera* (Chrysargyris et al., 2018; Rahneshan et al., 2018; Hajizadeh et al., 2021).

### 4.4 Phenolic and carotenoid dynamics (non-enzymatic antioxidant response)

Total phenolics increased to 4.5 mg GAE g^−1^ FW in S + PI (approximately 25% above the control) and 4.1 mg GAE g^−1^ FW in S + B, indicating improved antioxidant potential. A comparable phenolic–carotenoid co-regulation under salt stress was also observed. Carotenoid content also rose to approximately 3.2 mg g^−1^ FW under salinity, reflecting activation of photoprotective mechanisms. Combined treatments maintained moderate carotenoid levels (approximately 2.6–2.7 mg g^−1^ FW), suggesting optimized ROS control without excessive pigment accumulation. PCA and correlation analyses showed carotenoids clustering positively with RWC and phenolics but negatively with MDA, confirming coordinated antioxidant regulation. Similar metabolic coordination between phenolics and carotenoids under PGPR and osmolyte treatments has been documented in recent PGPR studies (Al-Turki et al., 2023), as documented by Lacerda et al. (2020), who linked secondary metabolite accumulation to improved ionic homeostasis in ornamentals. Similar increases in phenolic compounds contributing to enhanced antioxidant capacity under abiotic stress conditions have also been reported following silicon-based stress mitigation strategies (Ahsan et al., 2023).

### 4.5 Integrated biochemical response under salinity and biostimulant treatments

Multivariate analyses reinforced this physiological narrative. The PCA biplot (PC1 = 33.7% and PC2 = 25.0%, together explaining 58.7% of the total variance) illustrated coordinated regulation among oxidative, osmotic, and pigment traits, clearly separating treatments and visualizing their associations with key physiological and biochemical parameters (**Figure 7**). In the ordination space, proline, carotenoids, and RWC loaded in the same quadrant as chlorophyll a + b, while MDA pointed in the opposite direction, mirroring their negative correlations in the Pearson matrix. Treatment ordination separated S (high MDA, low RWC and pigments) from S + PI (elevated RWC and pigments), confirming that osmolyte–auxin supplementation repositioned plants toward a hydrated, pigment-preserving state. This integrative response aligns with Liu et al. (2024), who emphasized carotenoid- and phenolic-based non-enzymatic protection under salinity, and with Zhou et al. (2024), who highlighted signal transduction links between ionic balance and redox pathways. Comparable multivariate coordination between osmotic balance and antioxidant protection was also reported in *Zinnia elegans*, where exogenous putrescine enhanced antioxidant activity, potassium uptake, and pigment stability under NaCl stress (Mohammadi et al., 2024). These responses closely parallel the the osmolyte antioxidant interactions observed in the present study. Similar PCA-based clustering of stress-alleviated groups was observed in *Brassica napus* (Riaz et al., 2025). Furthermore, Marasco et al. (2024) demonstrated that PGPR-driven auxin modulation enhances osmolyte metabolism and salinity tolerance, supporting the mechanistic framework observed in the S + PI and S + B treatments. Likewise, Musazade et al. (2025) reported that auxin metabolism and signaling interact dynamically with osmolyte accumulation and antioxidant regulation, forming an integrated network that fine-tunes abiotic stress responses. Although the present study primarily focused on non-enzymatic antioxidant responses (MDA, proline, carotenoids, and phenolics), future research should integrate enzymatic antioxidant markers (e.g., SOD, CAT, POD) to provide a more complete understanding of redox regulation under salinity stress in *Gerbera*. These findings reinforce that exogenous Proline IAA application strengthens stress resilience through cross-talk between hormonal and redox-protective pathways. Similarly, Ozherelieva et al. (2025) found that biostimulant application in apple trees enhanced pigment stability, increased relative water content, and reduced lipid peroxidation by reinforcing proline–phenolic metabolism, mirroring the integrative osmotic–antioxidant regulation observed in *G. jamesonii*. Consistent multivariate evidence also comes from wheat under silicon supplementation, where PCA and correlation analyses separated salinity-only plants from Si-treated ones and linked higher RWC and chlorophyll/carotenoid levels with lower MDA and electrolyte leakage under NaCl stress (Singh et al., 2022). Collectively, Proline IAA and bacterial inoculation acted through complementary physiological pathways involving osmotic regulation, phenolic metabolism, and non-enzymatic ROS detoxification, ensuring pigment stability, membrane integrity, and enhanced salt tolerance in *G. jamesonii*.

### 4.6 Cultivar-specific morphophysiological responses

The two Gerbera cultivars responded to salinity in the same direction, but the severity of the response differed. Under NaCl stress, Sene Vidi showed greater reductions in plant height, leaf number and leaf area, confirming its higher sensitivity (Uzma et al., 2022a). Zingana maintained larger leaves and better overall growth, which aligns with studies in other crop species where tolerant cultivars preserve vegetative performance under salinity (Abdullah et al., 2022; Hameed et al., 2025). The mitigation treatments also separated the cultivars: Proline–IAA improved growth more clearly in Sene Vidi, while PGPR was more effective in Zingana, especially for leaf area and pigment retention (Acharya et al., 2024). Biochemical traits supported this distinction; Sene Vidi accumulated more MDA, whereas Zingana preserved chlorophyll and phenolics more consistently (Uzma et al., 2022b). PCA further reflected these differences, with Sene Vidi associating with proline and RWC and Zingana aligning with carotenoids and phenolics (Uzma et al., 2023). Overall, Sene Vidi coped mainly through osmotic adjustment, while Zingana relied more on pigment maintenance and antioxidant capacity.

## 5 Conclusions

Salinity stress induced by NaCl markedly disrupted the physiological and biochemical homeostasis of *Gerbera jamesonii*, leading to impaired water regulation, accelerated chlorophyll degradation, and intensified oxidative membrane damage. The application of Proline IAA effectively alleviated these detrimental effects by enhancing osmotic adjustment, maintaining relative water content, and stabilizing chlorophyll pigments. This treatment also reduced lipid peroxidation (MDA) and promoted the accumulation of non-enzymatic antioxidants such as carotenoids and phenolic compounds, thereby restoring cellular redox balance under saline conditions. Positive correlations among RWC, pigment stability, and antioxidant activity further confirm that Proline IAA strengthens salinity tolerance through coordinated osmotic and oxidative protection mechanisms. Overall, these findings highlight Proline IAA as a sustainable and eco-compatible biostimulant capable of maintaining physiological integrity and ornamental quality in greenhouse-grown *Gerbera*. Future studies should focus on elucidating the molecular and enzymatic pathways underlying this response and validating its long-term efficacy under commercial production systems.

## Data Availability

The original contributions presented in the study are included in the article/supplementary material. Further inquiries can be directed to the corresponding author.
